# Factors Shaping Public Perceptions of a Range of Robotic Technologies in Surgery: Cross-Sectional Web-Based Survey

**DOI:** 10.2196/64224

**Published:** 2025-11-20

**Authors:** Sarek Shen, Deborah Xie, Andy Ding, Lisa Zhang, Francis Creighton

**Affiliations:** 1 Department of Otolaryngology-Head and Neck Surgery, Johns Hopkins School of Medicine, 601 N Caroline Dr, Baltimore, MD, 21287, United States, 1 4109551932; 2 Department of Otolaryngology-Head and Neck Surgery, Ohio State University School of Medicine, Columbus, OH, United States

**Keywords:** perception, robotic surgery, semiautonomous robotic surgery, surgical extenders, safety, reliability, structural equation model, technology, acceptance, model, public, perceptions, cross-sectional, web-based, assessment, robot-assisted, surgery, robot, robotic technology, medical care

## Abstract

**Background:**

Within the surgical field, there has been an evolution in the application of robotic technology. Fully automatic robotic systems and augmented visualization tools are being introduced and may eventually replace existing surgical extenders such as the da Vinci surgical system. The literature on public perception of robotic surgery is growing, though specific drivers of these attitudes remain under investigation.

**Objective:**

The aim of this study is to investigate the underlying motivators of public perceptions toward robotic surgeries with varying levels of autonomy through a formal technology acceptance model.

**Methods:**

An online survey was distributed via the Amazon Mechanical Turk platform. Survey participants were provided definitions of a continuum of robotic technologies: robotic surgical extenders (technology without independent actions), semiautonomous robotic surgery (technology that provides guidance to the surgeon and requires surgeon input), and fully autonomous robotic surgery (technology that performs tasks autonomously without direct human interaction). The survey assessed overall attitudes toward each application of robotic technology in surgery and included questions delineating specific receptivity based on (1) perceived usefulness, (2) social risk, (3) time risk, (4) personal risk, and (5) reliability. A technology acceptance model was built to identify associations between these factors and overall attitudes toward robotic and semiautonomous surgeries.

**Results:**

A total of 1221 survey responses were recorded (mean age 38, SD 12 y; females: n=635, 52%). Individuals were more willing to accept robotic surgical extenders and semiautonomous robotic surgery compared to autonomous robotic surgery. Higher levels of education and better self-reported health were correlated with more positive attitudes toward autonomous robotic surgery. Perceptions of these technologies were not associated with age, gender, or income. Overall, attitudes toward robotic technologies in surgery were driven by views on the reliability, safety, and efficiency of the procedures. There was less concern regarding time risk and social risk associated with robotic and semirobotic surgeries.

**Conclusions:**

The public is more accepting of semiautonomous surgery and surgical extenders than fully autonomous surgery. General perceptions of the reliability, safety, and efficiency of these technologies drive variations in attitude. Time and social risk do not appear to have a significant impact on receptivity. Understanding these perspectives can help guide education within an advancing surgical field.

## Introduction

Applications of robotic and artificial intelligence technologies in surgery are rapidly developing [1-6]. The predominant robotic surgical technology currently available is surgical extenders such as the da Vinci (Intuitive)—these robotic technologies move only in response to surgeon input and provide nonaugmented visualization to the surgeon. Following surgical extenders, several semiautonomous systems have emerged in various surgical fields. At present, these technologies generally involve the incorporation of image guidance or augmented reality to identify anatomic structures and navigate an operative field [7,8]. Within orthopedics, semiautonomous systems are being used for improved hardware placement during spine surgery and hip surgery [9-13]. Looking further, researchers have developed fully autonomous robotic systems capable of performing tasks such as cochlear implantation [14-16] and laparoscopic bowel anastomosis [17,18]. Although not yet commercially available, these systems may emerge as standard complements to the surgical workflow.

Despite the promising developments within robotic surgery technologies, there remain social, financial, and regulatory barriers to these systems becoming commonplace. One obstacle is a nascent understanding of the public’s perception and tolerance of these technologies. Currently available robotic systems have been shown to improve operating room efficiency and surgical planning [19-23], and the public’s attitudes toward these technologies continue to evolve. There is a growing body of research investigating the relationship between sociodemographic factors and views on autonomous technologies within medicine [24,25]. However, the underlying drivers of these perceptions are not fully explored. Given the growing prevalence of autonomous and semiautonomous technologies in our day-to-day lives, we sought to investigate the public’s perspectives of the spectrum of robotic systems in surgery. Using a technology acceptance model, we investigate the public’s attitudes, and drivers of those attitudes, toward a range of robotic surgery technologies.

## Methods

### Ethical Considerations

This study was reviewed and approved by the Johns Hopkins institutional review board (IRB00267594). A survey was designed on Qualtrics (Qualtrics LLC) and distributed through Amazon Mechanical Turk (MTurk) in October 2020 (Multimedia Appendix 1). MTurk is a crowdsourcing platform that allows individuals to complete assignments online in an anonymous fashion. All study data were deidentified after collection was complete. A US $0.25 compensation was provided for those who completed the survey and correctly answered all attention check questions.

### Participant Recruitment

Respondents were included if they were over 18 years of age and a US resident. The survey included multiple attention check questions to ensure active engagement (eg, “Please select the number 3”). Respondents were excluded if their primary language was not English or if they failed to answer attention check questions correctly. We do not have data on the number of people who viewed but elected not to partake in the survey; therefore, we cannot calculate traditional participation rates. The completion rate of those who started the survey was 97.2%.

### Survey Instrument

The survey instrument was designed to assess perceptions and attitudes toward applications of robotic technology in surgery. Each survey started by providing the participant with definitions of 3 levels of robotic technology in surgery:

- Robotic surgical extenders: robot controlled via surgeon, does not perform actions independently without the input of the surgeon.
- Semiautonomous robotic surgery: provides guidance to surgeon based on a patient’s information. The robot does not perform actions independently without surgeon input.
- Autonomous robotic surgery: operates and performs tasks on its own, without direct interaction with the surgeon.

Questions in the survey were adopted from previously validated surveys assessing acceptance of technology [26-28]. Respondents rated statements on a 7-point Likert scale from “strongly disagree” to “strongly agree.” Higher scores indicated a more positive or favorable opinion toward the technology being assessed. A respondent’s overall attitude (A) was interrogated directly via questions in the survey. Further questions were designed to assess one of the following latent variables:

- Perceived usefulness (PU): The ability for robotic technology to improve the care that one receives in surgery.
- Social risk (SR): The possibility that using robotic technology in surgery would lead to disapproval from one’s friends, family, or social group.
- Time risk (TR): The loss of time and inconvenience that would result from the use of robotic technology in surgery.
- Personal risk (PR): The physical safety of robotic technology in surgery.
- Reliability (R): The ability of robotic technology in surgery to work reliably over time.

Each latent variable was evaluated by multiple questions, abbreviated by the question number and the latent variable assessed (eg, PU1 and PU2 were the two questions assessing perceived usefulness). All questions were completed for each application of robotic technology in surgery (ie, each latent variable was assessed for robotic surgical extenders, semiautonomous robotic surgery, and autonomous robotic surgery). The relationship between the latent variables and the overall attitude was determined with structural equation models (SEMs) based on the technology acceptance model [29] and theory of planned behavior [30,31]. Participants were also asked to rate their personal comfort and trust with technology, as well as their own health on a 5-point Likert scale. Demographic data of each respondent were also collected. The survey instrument is included in the Multimedia Appendix 1.

### Data Analysis

Descriptive analyses, including frequency distribution and central tendency, were performed to illustrate the demographic characteristics and participants’ questionnaire responses. Participant education and self-perceived health levels were further stratified into quartiles. ANOVA, *t* tests, and Pearson correlation coefficient were used to compare participants’ attitudes and intentions among the surgical extender types, as well as differences in viewpoints among demographic variables.

Confirmatory factor analysis was used to measure the relationship between the measured variables (question responses) and the latent variables (key factors) through factor loading. Multiple SEMs were built to identify associations between the latent variables and overall attitude. Standard regression coefficients calculated by the model represent the ability of our survey questions to capture data on its latent variable. The assumption of multivariate normality was assessed and confirmed with skewness and kurtosis tests. Cronbach α was computed for each key factor to determine the internal consistency of the research construct. Values of >0.7 indicate high reliability, between 0.5 and 0.7 indicate moderate reliability, and <0.5 indicate low reliability [32].

Root mean square error of approximation and *Χ*^2^ tests were used to measure the discrepancy between the model-based and observed correlation matrices. The final structural model was built and standard regression coefficients were calculated. Data analyses were performed with R (version 4.1.2; R Foundation for Statistical Computing), with SEM calculations performed using the *lavaan* package. All α levels and error probabilities were set at the standard .05 level.

## Results

### Background Characteristics

A total of 1221 surveys were included for analysis. The mean age of participants was 37.7 (SD 12.1) years. Females made up 51.7% (n=631) of respondents, and the majority (n=940, 77%) were White. Full demographics are listed in Table 1.

**Table 1.** Participant sociodemographics and personal health ratings.

Characteristics	Values
Age (years), mean (SD)	37.7 (12.1)
Gender, n (%)	
Male	580 (47.5)
Female	631 (51.7)
Nonbinary	10 (0.8)
Race/ethnicity, n (%)	
White	940 (77)
African/African American	124 (10.2)
American Indian/Alaska Native	37 (3)
Asian	91 (7.5)
Other	29 (2.4)
Highest level of education, n (%)	
High school graduate or less	78 (6.4)
Some college	226 (18.5)
College degree	671 (55)
Advanced degree (Master’s, PhD, MD, etc)	246 (20.1)
Personal rating of health, n (%)	
Excellent	266 (21.8)
Very good	533 (43.7)
Good	334 (27.4)
Fair	73 (6)
Poor	15 (1.2)

### Sociodemographic Associations

**Figure 1.**
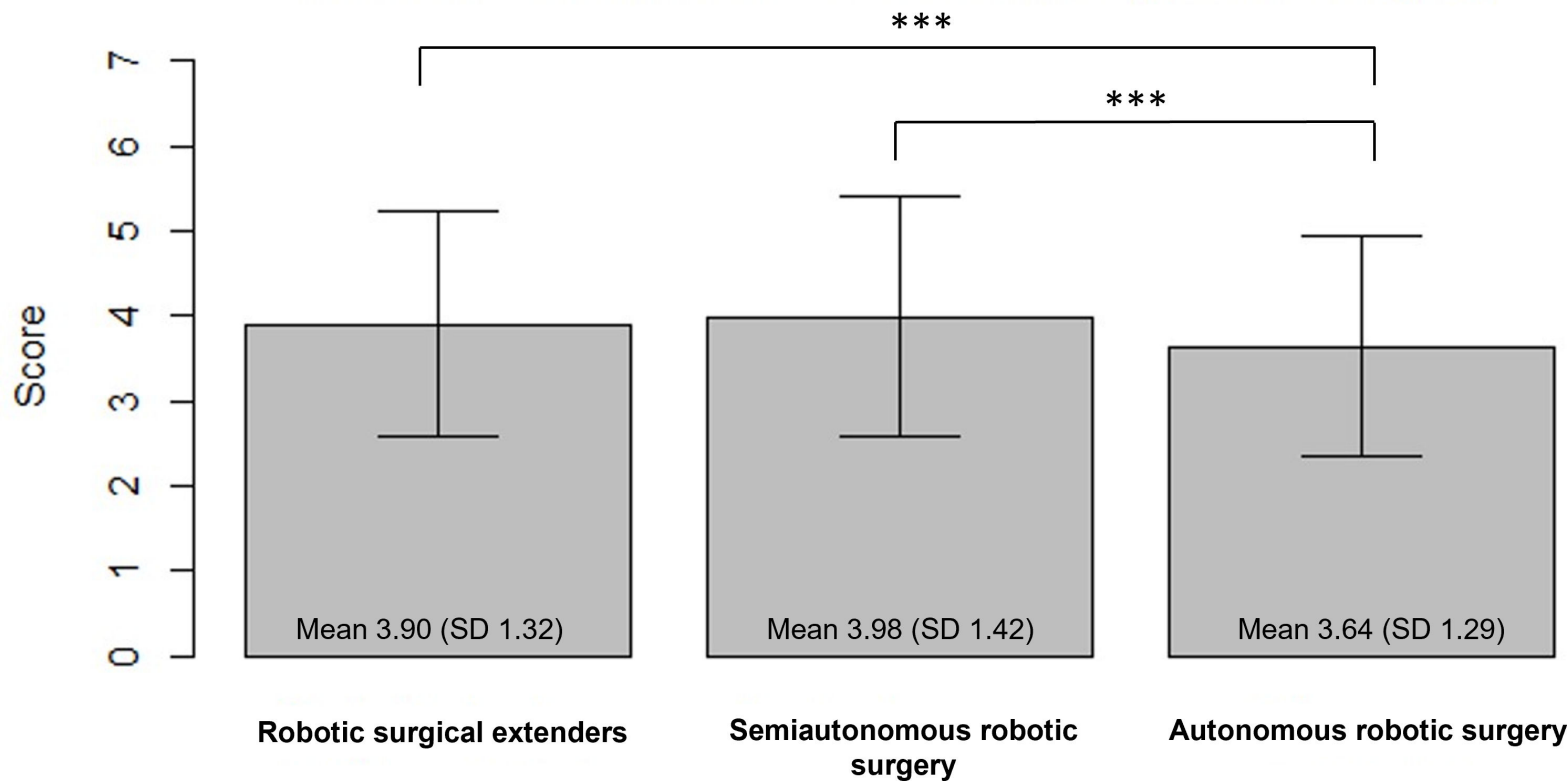
Comparison of overall attitudes toward different applications of robotic technology in surgery. ***P<.001.

The surveyed cohort expressed more positive attitudes toward robotic surgical extenders and semiautonomous robotic surgery compared to autonomous robotic surgery (Figure 1). There was no significant correlation between age and attitude toward robotic surgical extenders (*r*=0.024, 95% CI −0.032 to 0.080), semiautonomous robotic surgery (*r*=0.017, 95% CI −0.039 to 0.073), or autonomous robotic surgery (*r*=−0.0124, 95% CI −0.069 to 0.044). Similarly, there was no significant correlation between gender and race relative to attitude toward all applications of robotic technologies in surgery. There was a positive correlation between comfort with technology and overall attitudes for robotic surgical extenders (*r*=0.090, 95% CI 0.034 to 0.145), semiautonomous robotic surgery (*r*=0.088, 95% CI 0.032 to 0.143), and autonomous robotic surgery (*r*=0.118, 95% CI 0.062 to 0.173).

**Figure 2.**
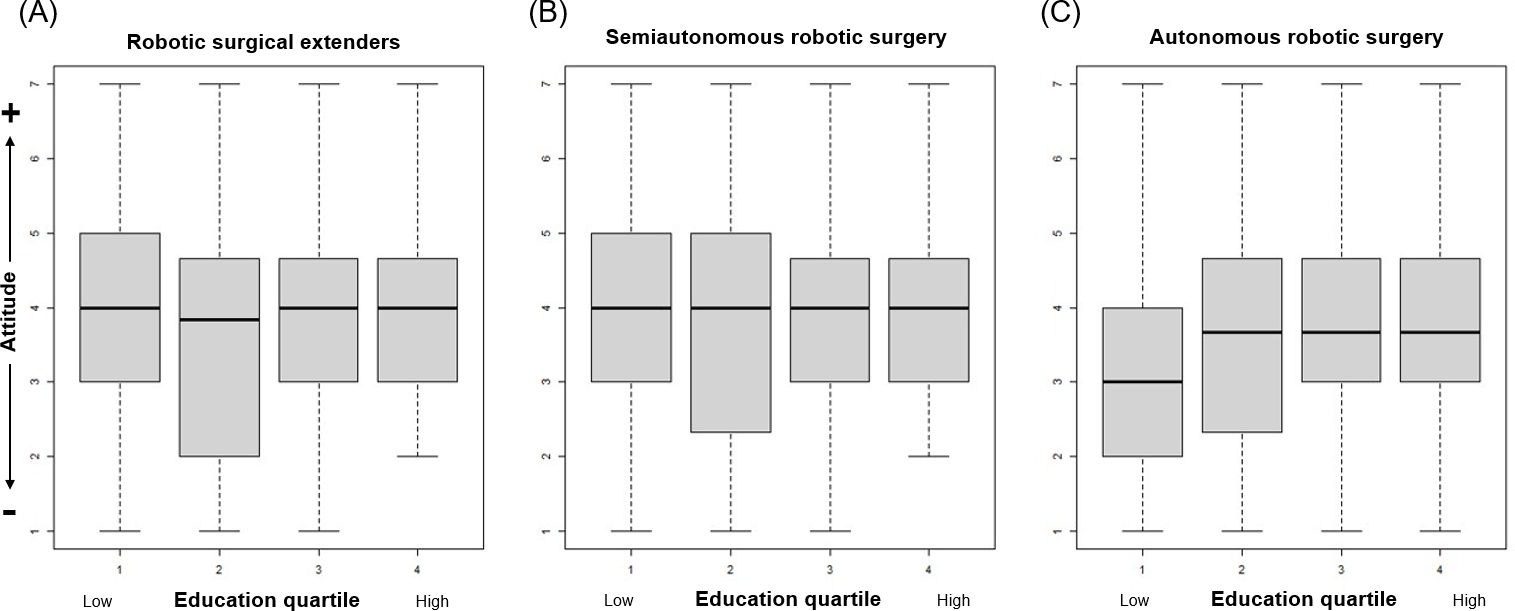
Relationships between education and overall attitudes toward different applications of robotic technology in surgery.

**Figure 3.**
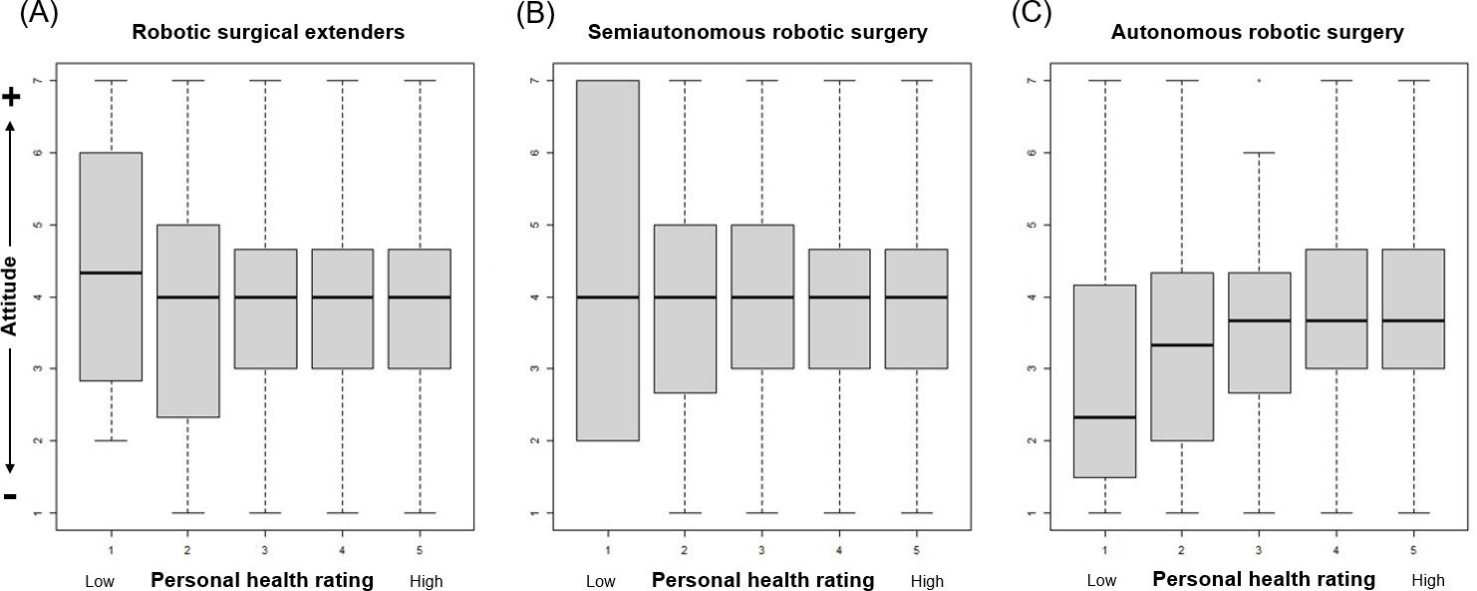
Relationships between personal ratings of health and overall attitudes toward different applications of robotic technology in surgery.

Attitudes toward robotic surgical extenders (*P*=.24) and semiautonomous robotic surgery (*P*=.57) did not vary based on level of education. However, respondents with higher levels of education did have more positive attitudes toward autonomous robotic surgery (*P*<.001, Figure 2). Similarly, there was no significant difference in attitudes toward robotic surgical extenders (*P*=.60) and semiautonomous robotic surgery (*P*=.29) across all levels of personal ratings of health. Those with higher ratings of health held more favorable attitudes toward autonomous robotic surgery (*P*=.003, Figure 3). There was no significant difference in attitudes toward all applications of robotic technology between individuals with or without a surgical history (all *P*>.05).

### Technology Acceptance Model  

**Figure 4.**
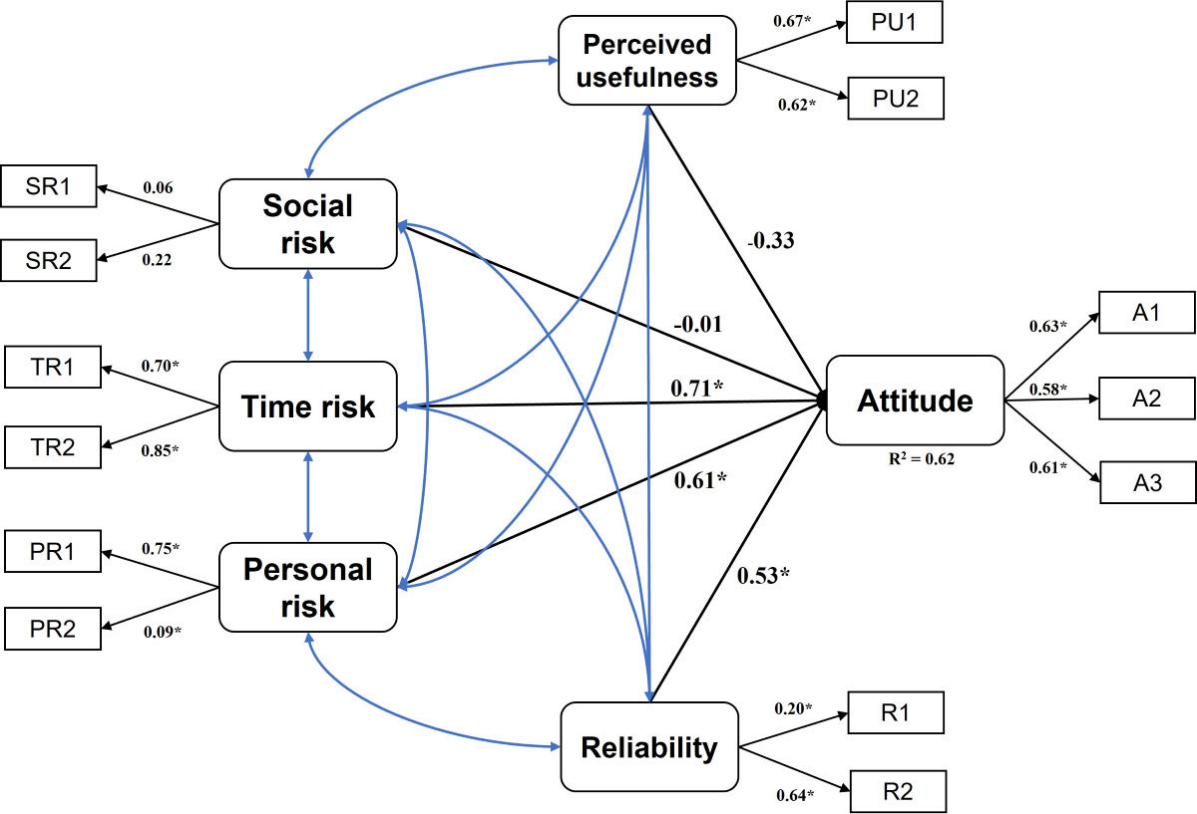
Results of structural equation modeling analysis. A: attitude; PR: personal risk; PU: perceived usefulness; R: reliability; SR: social risk; TR: time risk. *P<.05.

An SEM was built to explore the relationships between the latent variables (PU, SR, TR, PR, and R) and overall attitude (Figure 4). The correlation between survey questions for each latent variable was calculated using Cronbach α (Table 2). The low coefficient magnitude (SR1: 0.06, SR2: 0.22) combined with the lack of statistical significance suggest that the concept of “social risk” was poorly characterized by our survey questions. Attitudes toward robotic technologies in surgery were predicted by TR (*β*=0.71, *P*<.05), PR (*β*=0.61, *P*<.05), and R (*β*=0.53, *P*<.05). Together, these variables explain 62% of the variance of attitudes (*R*^2^=0.62, coefficient of determination). SR (*β*=−0.01, *P*>.05) and PU (*β*=−0.33, *P*>.05) did not significantly affect attitude.

**Table 2.** Cronbach α of the survey questions assessing the included latent variables (perceived usefulness, social risk, time risk, personal risk, reliability) and overall attitude.

	Robotic surgical extenders	Semiautonomous	Autonomous	Total
Perceived usefulness	0.81	0.46	0.26	0.59
Social risk	0.01	0.09	0.15	0.02
Time risk	0.76	0.8	0.68	0.75
Personal risk	0.01	0.16	0.34	0.22
Reliability	0.24	0.28	0.38	0.26
Attitude	0.46	0.52	0.45	0.63

## Discussion

### Principal Findings

Implementation of robotic technology in the operating room setting has the potential to improve the delivery of health care. From a surgeon’s perspective, the technical benefits of robotic surgery include improved visualization, better maneuverability, and favorable ergonomics [33,34]. As automated technologies become increasingly integrated into medicine, there has been a growing body of literature on how the public perceives the continuum of robotic procedures [35]. Palmisciano et al [19] found that patients are more favorable toward the use of autonomous technology in noninvasive applications, such as operative planning and preoperative imaging, compared to surgical procedures. In our study, we similarly found that the public is more open to surgical extenders and semiautonomous technologies than fully autonomous systems. Based on our technology acceptance model, the differences in these attitudes appear to be driven primarily by concerns involving safety and reliability, more so than social pressures or apprehension about the usefulness of the technologies. These findings may help guide efforts in patient education as these robotic systems become more commonplace in surgery.

### Sociodemographic Correlations

Our study found that individuals with higher levels of education, as well as those with higher personal ratings of health, tend to be more receptive toward fully autonomous surgeries. Education has been well-studied within the technology acceptance model; numerous investigations have demonstrated increased acceptance and utilization of technology among those with secondary or tertiary degrees [36-38]. Werner et al [39] have postulated that this is associated with a relative increase in access to and familiarity with new technologies, while Chen et al [40] have noted that these cohorts are more self-efficacious and may have less technology-related anxiety. From a health perspective, we found that participants with poorer self-ratings of health were associated with a more negative attitude toward fully robotic surgeries. This finding is congruent with studies investigating patterns of use of mobile health devices; for example, Chandrasekaran et al [41] showed that healthier patients are more likely to adopt new health care–associated technology [42]. However, these associations are complex and likely depend on the specific technology in question. Further study is necessary to elucidate the complicated relationship between the perceived health and technology acceptance of surgical patients.

Interestingly, we did not find a correlation between age and attitude toward fully autonomous surgeries. Recent studies investigating technology acceptance within aging populations, such as in Japan and South Korea, have similarly demonstrated a positive perception of robotic technology in an older cohort [43,44]. This is in part driven by the growing ubiquity of semiautonomous technology within day-to-day life, which increases familiarity, builds trust, and changes beliefs and attitudes toward new technology [45].

### Drivers of Public Perception

In investigating the drivers of overall patient attitude toward robotic surgeries, we found that risk to patient, reliability of technology, and perceived time risk were the primary concerns. There was less consideration for usefulness or social risk. These results are similar to those found in the autonomous automobile industry [46,47] and for assistive technology in patients undergoing physical rehabilitation [48]. Other studies have also found that apprehension toward adoption of robotic surgeries is driven by fears of equipment error or malfunction [34,49]. The evolution of attitudes toward autonomous driving may be particularly informative. Studies in this space suggest that increased system transparency, which refers to improved user understanding of the operational predictability, is positively associated with acceptance of the technology [46]. Within surgery, an increase in system transparency may begin with addressing misconceptions about these robotic surgical technologies. The colloquial definition and usage of the term “robot” tends to imply a programmable machine that can replicate the actions of a person. For those not familiar with robotic technology in health care, the nuances of its uses may not be readily obvious. In 2016, Boys et al [34] published a survey-based study specifically investigating the perceptions of robotic surgical extenders; interestingly, 21% of their 747 respondents believed that robotic surgical extenders had autonomous function during surgery. To address these misconceptions, the creation of accessible patient education materials, specifically focusing on reliability, safety, and efficiency, may be the best way to impact overall attitudes toward robotic technologies in surgery.

### Limitations

There are several limitations to this study. First, while questions in our instrument had been previously validated [26-28], the consistency of responses related to the latent variables, specifically the social risk, was lower than expected. This may reflect inherent difficulty in summarizing a broad variable within 2‐3 questions, particularly within health care. However, the drivers of patient attitude that our group identified are similar to those implicated in other areas with a growing autonomous technological presence, including online banking and self-driving cars [26,27,50]. This concordance is reassuring and suggestive of a similar pattern of technological acceptance between fields. Second, we understand that there may be a slight selection bias in the population sampled, as we used an online survey platform, which may have a younger and more technologically savvy population compared to the prototypical patient. However, it is notable that the mean age of patients undergoing the most common robotic procedure in the United States is around 41.5 years, consistent with our survey population. The racial and ethnic breakdown of patients undergoing robotic surgeries is similar to our survey cohort [51]. Additionally, prior studies have also shown that the Amazon MTurk survey results are comparable to those collected by more traditional methods [52-54]. Third, our survey was not able to capture additional socioeconomic variables, such as income or employment status, which may be hidden confounders of our findings. To address some of these limitations, future studies will include an in-person investigation into treatment decision-making and patient preference within robotic surgeries.

### Conclusion 

Current public perception toward surgical extenders and semiautonomous robotic surgery is more positive than that toward autonomous robotic surgery. Overall attitudes appear to be driven by concerns about the reliability, safety, and efficiency of these technologies. As robotic technologies emerge and continue to evolve within surgery, these findings can inform efforts in directed education. Comprehensive preoperative discussions targeted at the reliability and efficacy—rather than the usefulness or social risk—of these technologies may be more impactful. However, further work is needed in exploring potential obstacles to implementation of robotic systems within surgical practices.

## Supplementary material

10.2196/64224 Multimedia Appendix 1 Survey instrument

## Abbreviations

A: attitude
MTurk: Mechanical Turk
PR: personal risk
PU: perceived usefulness
R: reliability
SEM: structural equation model
SR: social risk
TR: time risk
