# Supplementary material for: Factors Shaping Public Perceptions of a Range of Robotic Technologies in Surgery: Cross-Sectional Web-Based Survey
Source: JMIR Hum Factors. 2025 Nov 20;12:e64224. doi: 10.2196/64224 (PMC12633834; doi:10.2196/64224)
Supplement: Multimedia Appendix 1 [file humanfactors-v12-e64224-s001.pdf]

Introduction

Purpose: This survey examines public preferences regarding robotic surgery. In the following study, you will be asked questions about your thoughts on robotic surgery. New robotic technology is being constantly developed, and some surgeons are using these technologies to assist in surgery. We want to better understand what people think of this. This study will take approximately 15-20 minutes to complete. All responses will be recorded confidentially and stored securely. Your completion of the survey will serve as your consent to be in this research study. Upon analyzing the data, all identifying information will be removed, and the data will be analyzed in aggregate to further protect the privacy of your responses.

Please do NOT take this survey if you are under the age of 18. We sincerely appreciate your participation!

Definitions

Robotic surgical extenders: The surgeon sits at a console. The robot is next to the patient. The surgeon moves all parts of the robot from the console, and the robot directly mirrors the actions and motions of the surgeon. The robot does not perform any actions without the input of the surgeon. (e.g., using a robotic system to reach a tumor in the back of the tongue).
Think of: driving a car without any lane assist or collision detection features.

Semi-autonomous robotic surgery: The robotic system provides guidance to the surgeon during surgery based on a patient's information. It does not perform actions without input of the surgeon. (e.g., a system that plans a knee replacement based on a patient's anatomy).
Think of: driving a car which has features such as lane assist, collision detection, etc.

Autonomous robotic surgery: The robot operates and performs tasks on its own as an autonomous surgeon. It can operate without direct interaction with a surgeon. (e.g., the robotic system prepares and designs a knee replacement surgery).
Think of: a self-driving car which does not need a person to control it.

Questions

Robotic surgical extenders

As a reminder, robotic surgical extenders involve the surgeon controlling all the motions of the robot. The robot does not perform any actions without the surgeon.

Think of: driving a car without any lane assist or collision detection features.

1. Using robotic surgical extenders in surgery will help my surgeon take care of me.

2. I think robotic surgical extenders are useful.

3. My friends and family would approve of my surgeon using robotic surgical extenders.

4. If I decided to have robotic surgical extenders used during my surgery and something went wrong, my friends/family would think less of me.

5. Using robotic surgical extenders in surgery would take a lot of time.

6. My surgeon would have to waste a lot of time fixing any errors with robotic surgical extenders during surgery.

7. I believe robotic surgical extenders would be dangerous.

8. I would feel safe if my surgeon was relying on robotic surgical extenders.

9. Robotic surgical extenders will work reliably over time.

10. Technology supporting robotic surgical extenders may not perform well and make errors.

11. I think using robotic surgical extenders is a good idea.

12. In my opinion, it is desirable to use robotic surgical extenders in surgery.

13. I agree with the application of robotic surgical extenders in surgery.

14. Given access to this, I would want my surgeon to use robotic surgical extenders if I needed surgery so that they could better take care of me.

15. I could see myself asking my surgeon to use robotic surgical extenders in surgery.

Semi-autonomous robotic surgery

As a reminder, semi-autonomous robotic surgery is where the robot provides guidance to the surgeon and can alert the surgeon about potential complications. The robot does not perform any actions without the surgeon.

Think of: driving a car which has features such as lane assist, collision detection, etc.

16. Using semi-autonomous robotic technology in surgery will help my surgeon take care of me.

17. I think semi-autonomous robotic surgery is useful.

18. My friends and family would approve of my surgeon using semi-autonomous robotic technology.

19. If I decided to have semi-autonomous robotic technology used during my surgery and something went wrong, my friends/family would think less of me.

20. Using semi-autonomous robotic technology in surgery would take a lot of time.

21. My surgeon would have to waste a lot of time fixing any errors with semi-autonomous robotic technology during surgery.

22. I believe semi-autonomous robotic surgeries would be dangerous.

23. I would feel safe if my surgeon was relying on semi-autonomous robotic technology.

24. Semi-autonomous robotic surgery will work reliably over time.

25. Technology supporting semi-autonomous robotic surgery may not perform well and make errors.

26. I think semi-autonomous robotic technology in surgery is a good idea.

27. In my opinion, it is desirable to use semi-autonomous robotic technology in surgery.

28. I agree with the application of semi-autonomous robotic technology in surgery.

29. Given access to this, I would want my surgeon to use semi-autonomous robotic technology if I needed surgery so that they could better take care of me.

30. I could see myself asking my surgeon to use semi-autonomous robotic technology in surgery.

Autonomous robotic surgery

As a reminder, autonomous robotic surgery is where the robot is able to operate and perform tasks without any input from the surgeon.

Think of: a self-driving car which does not need a person to control it.

31. Using autonomous robotic technology in surgery will help my surgeon take care of me.

32. I think autonomous robotic surgery is useful.

33. My friends and family would approve of my surgeon using autonomous robotic technology.

34. If I decided to have autonomous robotic technology used during my surgery and something went wrong, my friends/family would think less of me.

35. Using autonomous robotic technology in surgery would take a lot of time.

36. My surgeon would have to waste a lot of time fixing any errors with autonomous robotic technology during surgery.

37. I believe autonomous robotic surgeries would be dangerous.

38. I would feel safe if my surgeon was relying on autonomous robotic technology.

39. Autonomous robotic surgery will work reliably over time.

40. Technology supporting autonomous robotic surgery may not perform well and make errors.

41. I think autonomous robotic technology in surgery is a good idea.

42. In my opinion, it is desirable to use autonomous robotic technology in surgery.

43. I agree with the application of autonomous robotic technology in surgery.

44. Given access to this, I would want my surgeon to use autonomous robotic technology if I needed surgery so that they could better take care of me.

45. I could see myself asking my surgeon to use autonomous robotic technology in surgery.

Additional Questions

How confident are you generally in your ability to learn and use new technologies?

How would you rate your level of experience with technology in general?

How would you rate your overall level of trust toward technology?

In general, would you say your health is: Excellent, Very good, Good, Fair, Poor?

Have you or any of your family members/close friends ever had surgery?

What is your age?

What is your gender identity?

In which country do you currently reside?

In which state do you currently reside?

Choose one or more races you identify as: Asian, American Indian, African American, Native Hawaiian or Pacific Islander, Caucasian, Other.

What is the highest level of education you have completed or the highest degree you have received?

What is your approximate household income?
